# Outcomes of Traumatic Brain-Injured Patients With Glasgow Coma Scale < 5 and Bilateral Dilated Pupils Undergoing Decompressive Craniectomy

**DOI:** 10.3389/fneur.2021.656369

**Published:** 2021-05-25

**Authors:** Zhiji Tang, Ruijin Yang, Jinshi Zhang, Qianliang Huang, Xiaoping Zhou, Wenjin Wei, Qiuhua Jiang

**Affiliations:** Department of Neurosurgery, Ganzhou People's Hospital, Ganzhou, China

**Keywords:** decompressive craniectomy, bilateral dilated pupils, GCS, mortality, traumatic brain injury

## Abstract

**Objective:** Decompressive craniectomy (DC) plays an important role in the treatment of patients with severe traumatic brain injury (sTBI) with mass lesions and intractably elevated intracranial hypertension (ICP). However, whether DC should be performed in patients with bilateral dilated pupils and a low Glasgow Coma Scale (GCS) score is still controversial. This retrospective study explored the clinical outcomes and risk factors for an unfavorable prognosis in sTBI patients undergoing emergency DC with bilateral dilated pupils and a GCS score <5.

**Methods:** The authors reviewed the data from patients who underwent emergency DC from January 2012 to March 2019 in a medical center in China. All data, such as patient demographics, radiological findings, clinical parameters, and preoperative laboratory variables, were extracted. Multivariate logistic regression analysis was performed to determine the factors associated with 30-day mortality and 6-month negative neurological outcome {defined as death or vegetative state [Glasgow Outcome Scale (GOS) score 1–2]}.

**Results:** A total of 94 sTBI patients with bilateral dilated pupils and a GCS score lower than five who underwent emergency DC were enrolled. In total, 74 patients (78.7%) died within 30 days, and 84 (89.4%) had a poor 6-month outcome (GOS 1–2). In multivariate analysis, advanced age (OR: 7.741, CI: 2.288–26.189), prolonged preoperative activated partial thromboplastin time (aPTT) (OR: 7.263, CI: 1.323–39.890), and low GCS (OR: 6.162, CI: 1.478–25.684) were associated with a higher risk of 30-day mortality, while advanced age (OR: 8.812, CI: 1.817–42.729) was the only independent predictor of a poor 6-month prognosis in patients undergoing DC with preoperative bilateral dilated pupils and a GCS score <5.

**Conclusions:** The mortality and disability rates are extremely high in severe TBI patients undergoing emergency DC with bilateral fixed pupils and a GCS score <5. DC is more valuable for younger patients.

## Introduction

Traumatic brain injury (TBI), which is a significant public health issue, has become the main cause of trauma-related death and disability worldwide (1, 2). China has more cases of TBI than many other counties, making it almost impossible to implement a nationwide epidemiological investigation (3, 4). The treatments, rehabilitation therapy, and permanent sequelae associated with TBI impose a substantial economic burden on patients' families and have profound impacts on society (5, 6).

Decompressive craniectomy (DC), with or without the removal of the intracranial hematoma, plays a pivotal role in the treatment of patients with TBI with substantial mass lesions or uncontrolled elevation of the intracranial pressure (ICP) (7, 8). The latest edition of the relevant guidelines provides the indications for and approaches to DC in patients with intractable ICP that is refractory to conservative treatment (secondary DC) and/or various types of intracranial lesions (primary DC) (9).

Although DC has saved innumerable lives in the past decades, the high postoperative mortality and disability rates in severely injured patients are not only devastating to patients and their family members but also challenging for neurosurgeons (10–12). Some factors associated with a poor prognosis after DC have been identified, including the initial Glasgow Score Scale (GCS) and the pupillary status after trauma or on admission (13–17).

Nevertheless, it is unclear whether DC should be performed in all patients with acute intracranial hematoma with bilateral dilated pupils and a low GCS score (usually lower than five) on admission or in the early phase of resuscitation (18–21).

Thus, in the present retrospective study, we investigated the clinical outcomes in patients undergoing emergency DC who had bilateral dilated pupils and a GCS score lower than 5 and identified the risk factors for an unfavorable prognosis.

## Methods

### Patient Population

We performed this retrospective cohort study from January 2012 to March 2019 in Ganzhou People's Hospital, Jiangxi Province, China. During this period, 4,553 TBI patients were admitted to our department. After reviewing the medical records, we obtained the clinical information for 94 sTBI patients who underwent primary DC and had bilateral dilated pupils and a GCS score <5 at the time of the operation. In the early phase of resuscitation (usually <24 h), these patients with increasing intracranial hematoma, rapid neurological worsening, bilateral dilated pupils, and decreasing GCS score underwent DC as the first therapeutic procedure. Patients with posterior fossa injuries, complicated open injuries, severe underlying diseases, brain stem injuries, respiratory and circulatory failure, and incomplete clinical data were excluded from this study. According to our exclusion criteria, we excluded eight patients undergoing DC with bilateral dilated pupils and a GCS score <5, including two patients with posterior fossa hematoma, three with bilateral traumatic cerebral infarction (“black brain”) but a DC was strongly requested by their relatives, one with intracranial aneurysm, and two with an open craniocerebral injury.

### Ethics Approval

We were authorized by the Ethics Committee of Ganzhou People's Hospital to use the clinical data for this study.

### Surgical Treatment Protocol

DC was performed in patients in whom evidence of a mass lesion such as an intracranial hematoma was observed on computed tomography (CT) scans. Bilateral, unilateral or bifrontal craniectomy was performed based on the location of the hematoma. The craniectomy always extended from the temporal bone to the floor of the middle fossa. The dura was opened, and synthetic material or the temporalis fascia was used for duraplasty. Due to the urgency of DC (bilateral dilated pupils, low GCS score, presence of a mass lesion), an ICP monitor was not routinely inserted prior to surgery.

### Data Collection and Definition

All data, such as patient demographics, radiological findings, clinical parameters, and preoperative laboratory variables, were extracted from hospital medical charts and electronic medical records.

Patient characteristics included age, sex, and injury mechanism. Abrupt changes and neurological deterioration are usually noted after neurological examinations performed soon after admission. The GCS score and pupil dilation in the present study were measured at the time of DC. An injury severity score (ISS) was estimated at admission according to the standard created by Baker et al. (22). After reviewing each patient's computerized tomography (CT) scans and medical records, radiographic characteristics such as subdural hemorrhage, cerebral contusion, epidural hemorrhage, and subarachnoid hemorrhage were noted. We categorized the midline shift as <5, ≥5 and <10, and ≥10 mm. The status of the cistern at the basal level was defined as partially effaced or completely effaced (23). Biochemical parameters were also noted, including the preoperative hemoglobin (Hb) level, prothrombin time (PT), and other laboratory indicators. The values of these laboratory variables were dichotomized as normal or abnormal based on general clinical experience or the relevant literature (24–29).

### Outcome Variable and Groups

In the present study, we investigated the prognosis of patients with bilateral dilated pupils and a GCS score <5 who underwent primary DC, and we identified the factors associated with a poor prognosis. Thirty-day mortality and a poor 6-month prognosis after primary DC, which represent the short- and long-term prognoses, were the individual outcomes in this study.

Accordingly, the first outcome was 30-day mortality, and patients were categorized into a surviving group and a non-surviving group.

The secondary outcome measure was the Glasgow Outcome Scale (GOS) score at 6 months after DC; this scale is widely used to assess the quality of life (8). We classified the patients based on their GOS scores into groups with favorable or unfavorable outcomes. In our study, an unfavorable outcome was defined as a GOS score of 1 (dead) or 2 (vegetative state), whereas a favorable outcome was defined as a GOS score of 3 (severely disabled), 4 (moderately disabled), or 5 (recovered). The rationale for this categorization is that severe disability is usually deemed to be more acceptable than a vegetative state by the relatives of the patients.

### Statistical Analysis

Continuous variables with skewed distributions, including age and ISSs, are presented as medians and interquartile ranges, while categorical data are presented as numbers. The age at the time of injury was dichotomized using 39 years as the cutoff point; the cutoff value was determined by the maximization of the Youden index in the receiver operating characteristic (ROC) curve analysis. In the present study, we categorized the ISS as ≤29 or >29; the cutoff value was determined based on the Youden index. Each categorical variable was compared using chi-square tests.

To avoid missing potentially relevant predictive factors, variables that were nearly significant (*p* < 0.1) in univariate analyses were included in the multivariate analysis using forward LR selection to identify the independent predictors of 30-day mortality and a poor 6-month prognosis. Statistical significance was defined as *P* < 0.05, and odds ratios (ORs) with their 95% CIs were calculated.

The statistical analysis was performed with SPSS (version 20.0, IBM SPSS Statistics).

## Results

### Baseline Characteristics

Table 1 shows the baseline characteristics. Based on the inclusion and exclusion criteria of this study, 94 sTBI patients with bilateral dilated pupils and a GCS score <5 before undergoing primary DC were enrolled. The ages of the patients ranged between 2 and 82 years [median age at presentation was 44.5 years (IQR 31–57)] and included 80 males and 14 females. In this cohort, a fall was the most common mechanism of injury, accounting for 40.4% of all injuries (38/94). The other mechanisms were motorcycle accidents (33%, 31/94), motor vehicle accidents (23.4%, 22/94), and violent attacks (3.2%, 3/94). The median ISS was 32 (26–42). As shown in Table 1, preoperative hypoxia was observed in 58 (61.7%) patients.

**Table 1:** Comparison between survivors and non-survivors with Glasgow Coma Scale <5 and bilateral dilated pupils undergoing decompressive craniectomy.

	**Total (*n* = 94)**	**Survivors (*n* = 20)**	**Non-survivors (*n* = 74)**	***P*-value**
Age	44.5 (31–57)	33.5 (21.5–47.5)	47 (38–59)	
≤39 years	34	13	21	0.002
>39 years	60	7	53	
GCS at time of DC(IQR)				0.007
3	39	3	36	
4	55	17	38	
ISS (IQR)				0.058
≤29	39	12	27	
>29	55	8	47	
Sex				0.152
Male	80	15	65	
Female	14	5	9	
Subdural hemorrhage	81	14	67	0.018
Epidural hemorrhage	21	9	12	0.006
Subarachnoid hemorrhage	86	18	68	0.788
Basal cisterns (completely effaced)	75	15	60	0.548
Preoperative hypoxia (yes)	58	10	48	0.225
Preoperative PLT (<100 × 10^9^/L)	5	1	4	0.943
Preoperative PT (>14 s)	25	3	22	0.186
Preoperative INR (>1.2)	23	3	20	0.267
Preoperative FIB (<2 g/L)	68	11	57	0.051
Preoperative APTT (>36 s)	26	2	24	0.047
Preoperative anemia (Hb <10 g/dL)	8	1	7	0.526
Hyperglycemia (>200 mg/dL)	43	9	34	0.94
Hypocalcemia (<2.1 mmol/L)	39	6	33	0.24

*Chi-square tests for categorical variables*.

*APTT, activated partial thromboplastin time; CT, computed tomography; FIB, fibrinogen; GCS, Glasgow Score Scale; INR, international normalized ratio; ISS, Injury Severity Score; IQR, interquartile range; PLT, platelet; PT, prothrombin time*.

### Imaging and Laboratory Data

The intracranial abnormalities noted on the radiological imaging examinations were subarachnoid hemorrhage (91.5%, 86/94), subdural hemorrhage (86.2%, 81/94), cerebral contusion (85.1%, 80/94), and epidural hemorrhage (22.3%, 21/94). In all 94 patients, midline shift was observed; midline shifts that were <5, ≥5 and <10, and ≥10 mm were observed in 10 (10.6%), 21 (22.3%), and 63 (67.0%) patients, respectively. Seventy-five patients (79.8%) had effaced cisterns, and 19 patients (20.2%) had partially effaced cisterns.

Table 1 also lists the proportions of patients with abnormal values for each laboratory parameter. Preoperative anemia, hyperglycemia, and hypocalcemia occurred in 8 (85.1%), 43 (45.7%), and 39 (41.5%) patients, respectively. A platelet (PLT) count <100 × 10^9^/L, prothrombin time (PT) > 14 s, international normalized ratio (INR) > 1.2, fibrinogen (FIB) level <2 g/L, and activated partial thromboplastin time (aPTT) > 36 s, which were considered abnormal values, were observed in 5 (53.2%), 25 (26.6%), 23 (24.5%), 68 (72.3%), and 26 patients, respectively.

### Thirty-Day Mortality After Primary DC and Associated Risk Factors

In total, 74 patients (78.7%) died within 30 days of DC. Univariate analysis (Table 1) identified several factors significantly (*P* < 0.1) associated with 30-day mortality after primary DC in patients with bilateral dilated pupils and a GCS score <5, namely, age (*P* = 0.002), GCS score (*P* = 0.007), ISS (*P* = 0.058), subdural hemorrhage (*P* = 0.018), epidural hemorrhage (*P* = 0.006), FIB level (*P* = 0.051), and APTT (*P* = 0.047).

A predictive multivariate logistic regression model was developed to identify the risk factors for 30-day mortality (Table 2). In multivariate analysis, age (OR: 7.741, CI: 2.288–26.189), preoperative APTT (OR: 7.263, CI: 1.323–39.890), and GCS score (OR: 6.162, CI: 1.478–25.684) were associated with the risk of postoperative 30-day mortality in patients with bilateral dilated pupils and a GCS score <5. These are summarized in Table 2.

**Table 2:** Multivariate logistic regression analysis for 30-day mortality after primary DC.

**Variables**	**Multivariate results** **(logistic regression, forward: LR)**		
	***P*-value**	**OR**	**95% CI**
Age	0.001	7.741	2.288–26.189
Preoperative APTT	0.023	7.263	1.323–39.890
GCS	0.013	6.162	1.478–25.684

*APTT, activated partial thromboplastin time; GCS, Glasgow Score Scale; CI, confidence interval; OR, odds ratio*.

### Poor 6-Month Prognosis After Primary DC and Associated Risk Factors

Table 3 shows the clinical and radiological characteristics of the patients with GCS scores <5 and bilateral dilated pupils before DC who survived within 30 days of DC. Of the 20 survivors, four had a GOS score of 5, four had a GOS score of 4, two had a GOS score of 3, and 10 had a GOS score of 2 or 1 at the last follow-up examination. According to our definition of an unfavorable outcome, 84 patients (89.4%) had a poor outcome. Univariate analysis (Table 4) showed that the following variables were potential predictors of a poor 6-month prognosis after primary DC: age (*P* = 0.019), basal cistern status (*P* = 0.099), preoperative FIB level (*P* = 0.016), and preoperative APTT (*P* = 0.039). Multivariate analysis (Table 5) identified age (OR: 8.812, CI: 1.817–42.729) as the only independent predictor of a poor 6-month prognosis after primary DC in patients with preoperative bilateral dilated pupils and a GCS score <5.

**Table 3:** Clinical and radiological characteristics of survival patients with GCS <5 and bilateral dilated pupils before DC.

**No**.	**Age**	**Mechanism of injury**	**GCS**	**ISS**	**CT findings**	**GOS at 6 months after DC**
1	15	Motorcycle	4	26	SDH, CC, SAH	2
2	16	Motorcycle	4	33	EDH, CC, SAH	4
3	20	Motor vehicle	4	29	CC, SAH	1
4	21	Motorcycle	4	26	SDH, EDH, CC, SAH	2
5	21	Motor vehicle	4	26	SDH, EDH, CC, SAH	2
6	22	Motorcycle	4	50	SDH, CC, SAH	2
7	24	Motorcycle	4	26	SDH, CC, SAH	5
8	28	Motor vehicle	4	30	EDH, CC, SAH	4
9	31	Fall	3	17	SDH, CC, SAH	5
10	33	Fall	3	42	SDH, EDH, CC, SAH	1
11	34	Fall	4	26	EDH	5
12	37	Fall	3	25	SDH, CC, SAH	4
13	39	Fall	4	38	SDH, EDH, CC, SAH	4
14	40	Motor vehicle	4	26	SDH, CC, SAH	5
15	47	Fall	4	42	SDH, CC, SAH	2
16	48	Fall	4	42	EDH	2
17	48	Fall	4	25	EDH, CC, SAH	2
18	60	Motorcycle	4	25	SDH, SAH	3
19	63	Motorcycle	4	26	SDH, CC, SAH	1
20	69	Motorcycle	4	38	SDH, CC, SAH	3

*DC, decompressive craniectomy; CT, computed tomography; GCS, Glasgow Score Scale; GOS, Glasgow Outcome Score; ISS, Injury Severity Score; SDH, subdural hemorrhage; CC, cerebral contusion; SAH, subarachnoid hemorrhage; EDH, epidural hemorrhage*.

**Table 4:** Comparison between good and poor outcomes with Glasgow Coma Scale <5 and bilateral dilated pupils undergoing decompressive craniectomy.

	**Total (*n* = 94)**	**Good prognosis (*n* = 10)**	**Poor prognosis (*n* = 84)**	***P*-value**
Age (≤39 years)	34	7	27	0.019
GCS at time of DC(IQR)				0.145
3	39	2	37	
4	55	8	47	
ISS (IQR) (>29)	55	4	51	0.209
Sex				0.156
Male	80	7	73	
Female	14	3	11	
Epidural hemorrhage	21	4	17	0.156
Subarachnoid hemorrhage	86	9	77	0.858
Basal cisterns (completely effaced)	75	6	69	0.099
Preoperative hypoxia (yes)	58	5	53	0.421
Preoperative PLT (<100 × 109/L)	5	0	5	0.428
Preoperative PT (>14 s)	25	1	24	0.209
Preoperative INR (>1.2)	23	1	22	0.26
Preoperative FIB (<2 g/L)	68	4	64	0.016
Preoperative APTT (>36 s)	26	0	26	0.039
Preoperative anemia	8	0	8	0.308
Hyperglycemia	43	3	40	0.29
Hypocalcemia	39	3	36	0.435

*Chi-square tests for categorical variables*.

*APTT, activated partial thromboplastin time; CT, computed tomography; FIB, fibrinogen; GCS, Glasgow Score Scale; INR, international normalized ratio; ISS, Injury Severity Score; IQR, interquartile range; PLT, platelet; PT, prothrombin time*.

**Table 5:** Multivariate logistic regression analysis for poor 6-month prognosis after primary DC.

**Variables**	**Multivariate results** **(logistic regression, forward: LR)**		
	***P*-value**	**OR**	**95% CI**
Age	0.007	8.812	1.817–42.729

*CI, confidence interval; OR, odds ratio*.

## Discussion

Although the removal of mass lesions and DC were performed promptly, 78.7% of the patients died within 30 days, and 89.4% of the patients had a poor 6-month prognosis; the outcomes in these severe TBI patients were disappointing. This result was similar to those reported in the literature. Park et al. (30) performed ultra-early decompressive craniectomy in 127 severe TBI patients, and they found that the mortality rate was 82.2% in patients with GCS scores of 4 and 5. It is noteworthy that these patients had a high ISS [32 (26–42), overall on average], with complex injuries observed on neuroimaging and a predominance of falls and motorcycle accidents as the injury mechanism.

Primary DC, in which mass lesions and a large bone flap are removed in an early stage of trauma, is a critically important method in the treatment protocol (31). However, in view of the discouraging outcomes in patients with bilateral fixed dilated pupils and a low GCS score (usually <5), it is still unclear whether these patients should undergo DC. Over the course of 4 years, Jamous et al. (18) performed DC in 21 patients with bilateral pupil enlargement and a GCS score of 3. The follow-up results showed that all the patients died within 30 days after DC. The authors pointed out that the mortality rate of these patients was 100%. Therefore, DC merely reduced the intracranial pressure and prolonged the dying process. The authors of that study did not recommend invasive treatment for such patients to avoid aggravating their pain and the burden on their families. In a long-term case series, Gaétane et al. (14) found that bilateral fixed dilated pupils were significantly associated with excess mortality. They consequently encouraged clinicians to limit the performance of DC in patients with very severe TBI (initial GCS score <5 and bilateral fixed pupil dilation). However, according to a meta-analysis reported by John, a favorable prognosis is possible in patients with bilateral fixed dilated pupils if surgery is performed in selected patients (32). After conducting a long-term follow-up study (1–10.5 years), Thomale found that a lower GCS score did not necessarily indicate an unfavorable outcome, especially in pediatric patients. Some patients may achieve ideal rehabilitation if DC is performed in a timely manner (19).

In this retrospective study, we found that age, preoperative APTT, and the GCS score independently predicted 30-day mortality after primary DC in TBI patients with bilateral fixed dilated pupils and a GCS score <5, while age was the only independent predictor of a poor outcome at 6 months. Many of the univariant significant results would no longer be significant after multivalent analyses. This shows that logistic regression analysis can eliminate more confounding factors and highlight independent risk factors, which is the advantage of this method. We believe that age is a critical factor in determining the prognosis. In our study, we also observed that 39 years of age was the threshold above which both the 30-day mortality rate and the 6-month unfavorable prognosis rate significantly increased. Huang et al. observed a similar result. In their study, 40 years of age was a cutoff point for predicting a higher mortality rate after DC (33). After reviewing clinical data from 103 TBI patients treated with DC, Matthew noted that the outcome was age-dependent. They also found that the age group from 35 to 49 years old had significantly worse GOS scores (34). We believe that neuroplasticity and tolerance of the secondary insults of TBI, such as traumatic brain edema, ischemia, and hypoxia, are lower in elderly patients than in young patients, which makes it difficult for elderly patients to achieve a favorable long-term outcome after DC.

Unfortunately, only 20 patients in our study survived within 30 days of DC, 10 of whom had poor long-term outcomes. We defined an unfavorable outcome as a GOS score of 1 or 2, which was slightly different from other studies. We found that, in our country, the minimum level of recovery needed for family members to accept the outcome is whether the patients are conscious after DC, even if they are seriously disabled. In contrast, the outcomes of death and persistent vegetative status are considered unacceptable. Therefore, we should reassess the value of DC in patients, especially elderly patients with bilateral fixed pupils and low GCS scores. Honeybul indicated that, in some cases, these patients who have experienced serious trauma have severe disabilities or are in a vegetative state. However, society determines the value of human existence, making it important to reexamine whether these outcomes are meaningful and acceptable to these patients and their families (21). In clinical practice, faced with substantial pressure from desperate family members, we must find a balance between ethics, emotions, and limited medical resources. Although the results of DC are not satisfactory, these poor outcomes are not the fault of the surgeons. We recommend that DC should be performed in young patients with bilateral fixed pupils and a lot GCS score because only those patients are likely to benefit from DC.

## Conclusions

The mortality and disability rates are extremely high in severe TBI patients undergoing DC with bilateral fixed pupils and a GCS score <5. DC is more valuable for younger patients.

## Data Availability Statement

The raw data supporting the conclusions of this article will be made available by the authors, without undue reservation.

## Ethics Statement

Written informed consent was obtained from the individual(s), and minor(s)' legal guardian/next of kin, for the publication of any potentially identifiable images or data included in this article.

## Author Contributions

QJ designed this study. ZT, JZ, and QH performed data collection. WW conducted statistical analysis. ZT and RY drafted the manuscript and contributed equally to the article. XZ reviewed the manuscript before submitting. All authors contributed to the article and approved the submitted version.

## Conflict of Interest

The authors declare that the research was conducted in the absence of any commercial or financial relationships that could be construed as a potential conflict of interest.
